# Hilar mossy cell oxytocin receptor signaling regulates adult hippocampal neurogenesis and context discrimination in mice

**DOI:** 10.1186/s12929-026-01221-6

**Published:** 2026-02-12

**Authors:** Yu-Chieh Hung, Yi-Yu Hsieh, Kuei-Sen Hsu

**Affiliations:** 1 Institute of Basic Medical Sciences, College of Medicine, National Cheng Kung University, Tainan, 70101 Taiwan; 2 Department of Pharmacology, College of Medicine, National Cheng Kung University, No. 1, University Rd., Tainan, 70101 Taiwan

**Keywords:** Oxytocin receptor, Mossy cell, Neural stem/progenitor cells, Adult neurogenesis, Context discrimination, Hippocampus

## Abstract

**Background:**

Neurogenesis is a crucial form of neuroplasticity in the adult mammalian brain. Hilar mossy cells (MCs) in the dentate gyrus show a uniquely high expression of oxytocin receptors (OXTRs) and are implicated in the regulation of adult hippocampal neurogenesis (AHN). However, it remains unclear whether MCs regulate AHN through OXTR signaling. Here, we sought to investigate whether loss of MC OXTR signaling affects AHN and its associated cognitive function.

**Methods:**

We used a calcitonin receptor-like receptor (*Crlr*)-Cre mouse line to selectively remove *Oxtr* in MCs. 5-Bromo-2'-deoxyuridine labeling, immunofluorescence staining, and retrovirus-mediated strategies were used to trace newborn cells. The contextual fear discrimination task was employed to evaluate learning and memory functions mediated by AHN.

**Results:**

We found that conditional deletion of MC *Oxtr* impairs AHN by reducing the number, but not the rate, of proliferation, differentiation, survival, and maturation of neural stem/progenitor cells (NSPCs). MC *Oxtr* deletion reduces cell cycle re-entry and promotes cell cycle exit and NSPC death. Furthermore, MC *Oxtr* deletion reduces the populations of type 1, type 2b, and type 3 NSPCs. Using a retrovirus-mediated birthdating and cell-labeling approach, we demonstrate that deletion of MC *Oxtr* retards dendritic development without affecting the migration or positioning of newly generated dentate granule cells. Functionally, MC OXTR-deficient mice exhibited impaired performance in the contextual fear discrimination task, indicating a deficit in fear memory specificity.

**Conclusions:**

These results uncover a previously unknown role for MC OXTR signaling in regulating the dynamic processes of AHN and context discrimination.

## Background

In the dentate gyrus (DG) of the adult hippocampus, neural stem/progenitor cells (NSPCs) are maintained beyond development within a specialized neurogenic niche where newborn neurons are generated and integrated into the preexisting neural network throughout life in mammals, including humans [1–4]*.* This process, known as adult hippocampal neurogenesis (AHN), has been linked to various aspects of cognitive function, including learning, memory, pattern separation, and cognitive flexibility, as well as the behavioral impacts of stress and antidepressants [3, 5]. AHN is a complex, multistep process dynamically regulated by intrinsic and extrinsic factors under physiological and pathological conditions [1, 6]. Besides identifying multiple molecular factors and intracellular signaling pathways involved in regulating AHN, there is growing awareness of the roles of local neural circuit activity and long-distance projections in regulating AHN [7–12]. Mossy cells (MCs) are a major population of excitatory neurons located in the hilus, regulating DG granule cell (DGC) activity directly via monosynaptic excitatory connections or indirectly via GABAergic inputs [13–15]*.* Interestingly, MCs provide both the initial glutamatergic synaptic inputs and disynaptic GABAergic inputs onto newly generated DGCs [16–18]. Surprisingly, there have been few studies to date that explore the role of MCs in regulating AHN. A previous study demonstrated that within the DG neurogenic niche, MCs regulate the fate of NSPCs through a dynamic balance between direct glutamatergic and indirect GABAergic pathways, and chronic ablation of MCs results in transient activation of NSPCs followed by pool depletion [11]. Another recent study reported that hilar MC activity is required for behavioral and neurogenic responses to chronic antidepressant treatment [19]. Nonetheless, whether and how MCs regulate the dynamic processes of AHN, from the activation of quiescent NSPCs to the integration of neural networks and the survival of newly generated DGCs, awaits further investigation.

The hypothalamic neuropeptide oxytocin (OXT) is traditionally recognized for its role in modulating mammalian social behaviors, including maternal care, affiliation, pair bonding, and social recognition [20, 21]. Notably, our previous study demonstrated that endogenous OXTR signaling can regulate AHN through an indirect non-cell-autonomous mechanism involving OXT receptors (OXTRs) expressed in hippocampal CA3a pyramidal neurons [22]. Consistently, chronic intranasal administration of OXT has been shown to enhance AHN [23]. In addition to hippocampal CA3a pyramidal neurons, we and others observed enriched OXTR expression in hilar MCs [24–26]. Additionally, our recent study highlighted that MC OXTR signaling is important for controlling the MC-GC network excitability in the DG [26]. However, whether MC OXTR signaling regulates AHN and related brain functions remains unclear. Here, to address this question, we employed a calcitonin receptor-like receptor (*Crlr*)-Cre mouse line to selectively ablate *Oxtr* in MCs. Our results reveal that MC OXTR signaling plays a critical role in regulating the proliferation, differentiation, survival, and maturation of NSPCs. MC OXTR signaling regulates dendritic growth of newly generated DGCs. Importantly, MC *Oxtr* deletion reduces NSPC populations and impairs contextual fear discrimination. Altogether, our data reveal a novel role of MC OXTR signaling in regulating AHN and context discrimination abilities.

## Materials and methods

### Animals

All experiments were conducted with approval (No. 113038) from the Institutional Animal Care and Use Committee at National Cheng Kung University and adhered to the National Institutes of Health (NIH) Guidelines for the Care and Use of Laboratory Animals. Homozygous *Oxtr*-floxed (*Oxtr*^f/f^) mice were purchased from Jackson Laboratory (Strain #: 008471, RRID: IMSR_JAX:008471), and heterozygous *Crlr*-Cre transgenic mice (RRID: IMSR_JAX:023014) were originally provided by Dr. Kazu Nakazawa (SRI) and Dr. Cheng-Chang Lien (NYCU). We crossed *Crlr*-Cre mice with *Oxtr*^J/J^ mice to generate MC-specific *Oxtr* cKO mice on a C57BL/6 genetic background [26]. Ai14 mice (Strain #: 007908, RRID: IMSR_JAX:007908) were crossed with *Crlr*-Cre or *Ascl1*-CreERT2 (Strain #:012882, RRID: IMSR_JAX:012882) mice for the generation of *Crlr-*Cre::Ai14 or *Ascl1*-CreERT2::Ai14 mice. To achieve indelible expression of tdTomato in newly generated DGCs of *Ascl1*-CreERT2::Ai14 mice, mice were injected intraperitoneally with 4-hydroxytamoxifen (120 mg/kg; Sigma-Aldrich, Cat#H6278) twice daily for 2 consecutive days, with a 12-h interval between injections, followed by a 14-day chase. Mice were genotyped by the polymerase chain reaction (PCR) method with genomic DNA extracted from tail samples. Experiments were conducted using 12–14-week-old male mice. Mice were housed in groups of 4 per cage in a temperature-controlled (25 ± 1 °C) and humidity-controlled (50 ± 5%) environment, following a 12 h:12 h light/dark cycle (lights on at 7:00 AM) with ad libitum access to food and water. All experimental procedures were performed during the light phase of the cycle. The investigators were blinded to treatment allocation during experiments and outcome assessments.

### Slice preparations for immunohistochemistry

Mice were anesthetized with zolazepam (50 mg/kg) plus xylazine hydrochloride (5 mg/kg) and transcardially perfused with 4 °C phosphate-buffered saline (PBS), followed by 4% paraformaldehyde (PFA) in 0.1 M PBS at pH 7.4 for fixation. Brains were removed, post-fixed overnight in 4% PFA at 4 °C, and then cryoprotected in a 30% sucrose solution at 4 °C for 48 h. The dorsal DG of the hippocampus, ranging from Bregma − 1.0 mm to − 2.3 mm, and the ventral DG, spanning Bregma − 2.4 mm to − 4.0 mm, were sectioned coronally at 40 μm thickness using a sliding microtome (Leica Microsystems, Wetzlar, Germany) for immunohistochemistry.

### BrdU labeling, immunofluorescence staining, and quantification of NSPCs

Fluorescent immunolabelling and stereological quantification of NSPCs were performed as previously described [22, 27]. To analyze progenitor cell proliferation, mice were injected with a single pulse of 5-bromo-2'-deoxyuridine (BrdU; 50 mg/kg; Sigma-Aldrich, St. Louis, MO) and analyzed 2 h later. To quantify neuronal differentiation, mice were injected intraperitoneally with BrdU twice daily for 3 consecutive days, with a 12-h interval between injections, followed by a 14-day chase. To quantify the survival and maturation of newly generated DGCs, mice were injected intraperitoneally with BrdU twice daily for 3 consecutive days, with a 12-h interval between injections, followed by a 28-day chase period. To analyze progenitor cell cycling, mice were given a single pulse of BrdU, and the number of cells that exited the cell cycle and then re-entered within 24 h after injection was measured.

Fluorescent immunolabeling was used to count the number of NSPCs. For BrdU^+^Ki67^+^, BrdU^+^doublecortin (DCX)^+^, BrdU^+^neuronal nuclear protein (NeuN)^+^, BrdU^+^calbindin (Calb)^+^, BrdU^+^SRY-box transcription factor 2 (SOX2)^+^glial fibrillary acidic protein (GFAP)^+^, and BrdU^+^SOX2^+^DCX^+^ labeling, free-floating sections were denatured in 10 mM saline-sodium citrate buffer at 85 °C for 30 min and then incubated at 37 °C for 30 min in 1 N HCl. Sections were rinsed twice for 10 min at 25 °C in 0.1 M Na borate (pH 8.5) and then incubated in the primary antibodies against OXTR (1:100; Alomone Labs, Cat#AVR013, RRID:AB_2651123), BrdU (1:500; Millipore, Cat#MAB4072, RRID: AB_95024), Ki67(1:1000; Abcam, Cat#ab15580, RRID:AB_443209), DCX (1:1000; Cell Signaling Technology, catalog#: 4604, RRID: AB_561007), NeuN (1:2000; Millipore, Cat#ABN78, RRID: AB_10807945), Calb (1:500; Millipore, Cat#AB1778, RRID: AB_2068336), GFAP (1:1000; Invitrogen, Cat#13–0300, RRID: AB_2532994) or SOX2 (1:1000; Abcam, Cat#ab97959, RRID: AB_2341193) overnight at 4 °C in PBS with 0.3% Triton X-100 and 30% bovine serum albumin. Finally, sections were washed in PBS with 0.4% Triton and then incubated with the Alexa Fluor 405-, 488- or 568-conjugated secondary antibodies [Thermo Fisher Scientific, Waltham, MA, Cat#A48255 (RRID: AB_2890536), Cat#A-11073 (RRID: AB_2534117)/Cat#A-11034 (RRID: AB_2576217)/A-11001 (RRID: AB_2534069), Cat#A-11075 (RRID: AB_141954)/Cat#A-11036 (RRID: AB_10563566)/Cat#A-11004 (RRID: AB_2534072)/A-11077 (RRID: AB_2534121)] for 2 h at room temperature. Cell nuclei were counterstained with 4',6-diamidino-2-phenylindole (DAPI, Abcam, Cat#ab104139). Upon completion of immunostaining, sections were collected on separate gelatin-subbed glass slides, rinsed extensively in PBS, and mounted with Fluoromount-G™ Mounting Medium (Invitrogen, Cat#00–4958-02, RRID: SCR_015961).

Cell death was identified using a primary antibody against cleaved caspase 3 (1:1000; Cell Signaling Technology, Cat#9579, RRID: AB_10897512), along with DAPI (1:5000; Sigma-Aldrich, Saint Louis, MO; Cat#D9542). GFAP (1:1000; Invitrogen, Cat#13–0300, RRID: AB_2532994) and cleaved caspase 3 were utilized to label the cell death of type 1 NSPCs in the SGZ.

Stereological quantification of BrdU-labeled cells was performed as previously described [22, 27]. The fluorescence intensity of BrdU^+^ cells was defined as at least twice that of the background level. Every sixth section covering the entire DG of the hippocampus was processed for BrdU immunohistochemistry. All BrdU-labeled cells in the granule cell layer, subgranular zone (SGZ), and hilus were counted under fluorescent illumination at 400 × using an Olympus BX51 microscope coupled to an Olympus DP70 digital camera. Total cell numbers were calculated by multiplying the counted cells in every sixth section by six. Fluorescence microscopic images were obtained using an Olympus FluoView confocal microscope (Olympus Corporation, Tokyo, Japan).

The balance between re-entry and exit of the cell cycle was assessed using BrdU/Ki67 immunolabeling as previously described [27, 28]. To assess the extent of NSPC populations, the numbers of BrdU^+^GFAP^+^SOX2^+^ (type 1), BrdU^+^GFAP^−^SOX2^+^ (type 2a), BrdU^+^SOX2^+^DCX^+^ (type 2b), and BrdU^+^SOX2^−^DCX^+^ (type 3) cells were counted. The proliferating neuroblasts were identified by BrdU and Ki67 expression. Neuronal differentiation was assessed by evaluating the expression of BrdU and DCX. Cell survival and maturation were determined by counting the number of BrdU + NeuN + and BrdU^+^Calb^+^ cells, respectively. All images were imported into NIH ImageJ (RRID: SCR_001935) for analysis, and all parameters used were kept consistent throughout image capture.

### Retrovirus-mediated labeling

Engineered self-inactivating murine retroviruses expressing green fluorescent protein (GFP) were used to label proliferating cells and their progeny in the DG of adult mice as previously described [22, 29]. High titers of engineered retroviruses (1 × 10^9^ units/ml) were produced by co-transfection of retroviral vectors and VSV-G into HEK293GP cells, followed by ultracentrifugation of viral supernatant. The Cre recombinase (Addgene, plasmid#20,781) was cloned into a retroviral expression vector Ubi-X-2A-GFP.

Experimental mice were anaesthetized with a mixture of zolazepam (50 mg/kg) and xylazine hydrochloride (5 mg/kg) and the purified retroviruses were stereotaxically injected into the DG at 4 sites (0.5 μl per site at 0.25 μl/min) with the following coordinates (anterior–posterior = − 2 mm from bregma, lateral ± 1.6 mm, ventral = 2.5 mm; anterior–posterior 3 mm from bregma, lateral =  ± 2.6 mm, ventral = 3.2 mm). Mice were sacrificed at 14 and 28 days post-infection (dpi) for morphological analysis. Coronal brain Sects. (40 μm) were prepared from virus-injected mice and processed for immunostaining as previously described [22, 29]. The sections were incubated in DAPI for 30 min before being washed and mounted. Images were acquired on an Olympus FluoView FV4000MPE multiphoton confocal system using a multi-track configuration. A z-stack of confocal images was acquired, and a single confocal slice with the largest soma area for each GFP^+^ neuron was selected for quantitative morphological analysis in the NIH ImageJ program. Dendritic complexity of neurons was measured using Sholl analysis, which counts the number of dendrites intersecting concentric circles at 10 μm intervals from the cell soma.

### Fluorescent in situ hybridization (FISH)

FISH analysis was conducted using the RNAscope® Multiplex Fluorescent Reagent Kit 2.0 (Advanced Cell Diagnostics) as previously described [22, 26]. Briefly, coronal brain slices (16 µm) were fixed in 4% PFA for 15 min and dehydrated in a series of 50%, 70%, and 100% ethanol for 5 min each. Sections were subjected to reagent Pretreat 3 at 25 °C for 30 min and then hybridized with probes at 40 °C for 2 h in a humidified oven (ACD HybEZ™ Hybridization System). The *Oxtr*-O1 probe (Cat# 454,011) and the *Gria2* probe (Cat#416,091-C2) were used to target *Oxtr* mRNA and *GluR2* mRNA, respectively. The *Oxtr*-O1 probe specifically targets exon 3 of the *Oxtr* mRNA. After hybridization, brain sections were sequentially applied with a series of probe signal amplification steps, washed with ACD Wash Buffer 2 times, 2 min each, and mounted with VECTASHIELD antifade mounting medium (Vector Laboratories) containing DAPI (1:5000; Sigma-Aldrich, Saint Louis, MO; Cat#D9542) for staining DNA.

### Contextual fear discrimination task

The contextual fear discrimination task was conducted using a computer-controlled context conditioning system (ENV-307A, MED Associates, Georgia, VT) as previously described [27]. Mice were trained to distinguish between two similar contexts, A and B, through repeated exposure to each context. The conditioning context A (15.9 × 14.0 × 12.7 cm) consisted of a stainless steel grid floor, aluminum side walls, a clear Plexiglas front door, and a white back wall. The chamber was indirectly illuminated by a 12-W light bulb. The context B was identical to context A, except for 4–5 rows of black spots on the three walls and the stainless-steel mesh floor. Each context was thoroughly sanitized with 70% ethanol after each trial to prevent olfactory cue bias. During the first three days, mice were placed in context A for 3 min to explore their environment. Following this exploration, they received a single footshock (0.65 mA for 2 s) and were returned to their home cage 1 min after the footshock. Afterward, the mice were trained to distinguish between the two contexts by being placed in each context daily, in a pseudorandomized order, with a 6-h interval between placements. This training lasted 10 days, from Day 4 to Day 13, as part of the discrimination task. Mice were subjected to a footshock 3 min after being placed in context A, but not in context B. Freezing behavior was measured as the total time spent freezing in each context during the first 3 min after the mice were placed in contexts A and B. Significant motion pixel (SMP) values measure motion linearly between frames captured at a rate of 7.5 Hz. The freezing threshold was defined as SMP < 20 for at least 1 s using Freezeview software (MED Associates). The discrimination ratio was calculated as (freezing in context A—freezing in context B)/(freezing in context A + freezing in context B).

### Statistical analysis

Statistical analyses were performed using GraphPad Prism 6 software. Sample sizes were chosen based on similar previous studies of a similar nature [22, 27]. No specific randomization method was used. Two-tailed unpaired Student’s *t*-test was used to compare differences between two independent groups. For non-normal distributions, we calculated *P* values using the Mann-Whitney *U*-test. Comparisons among more than two groups were analyzed using a two-way ANOVA with Sidak’s multiple comparisons test. All the data are presented as the mean ± SEM. Statistical significance was defined as *P* < 0.05. The statistical results are detailed in the figure legends.

## Results

### OXTR expression on hilar MCs but not NSPCs of the DG

**Fig. 1:**
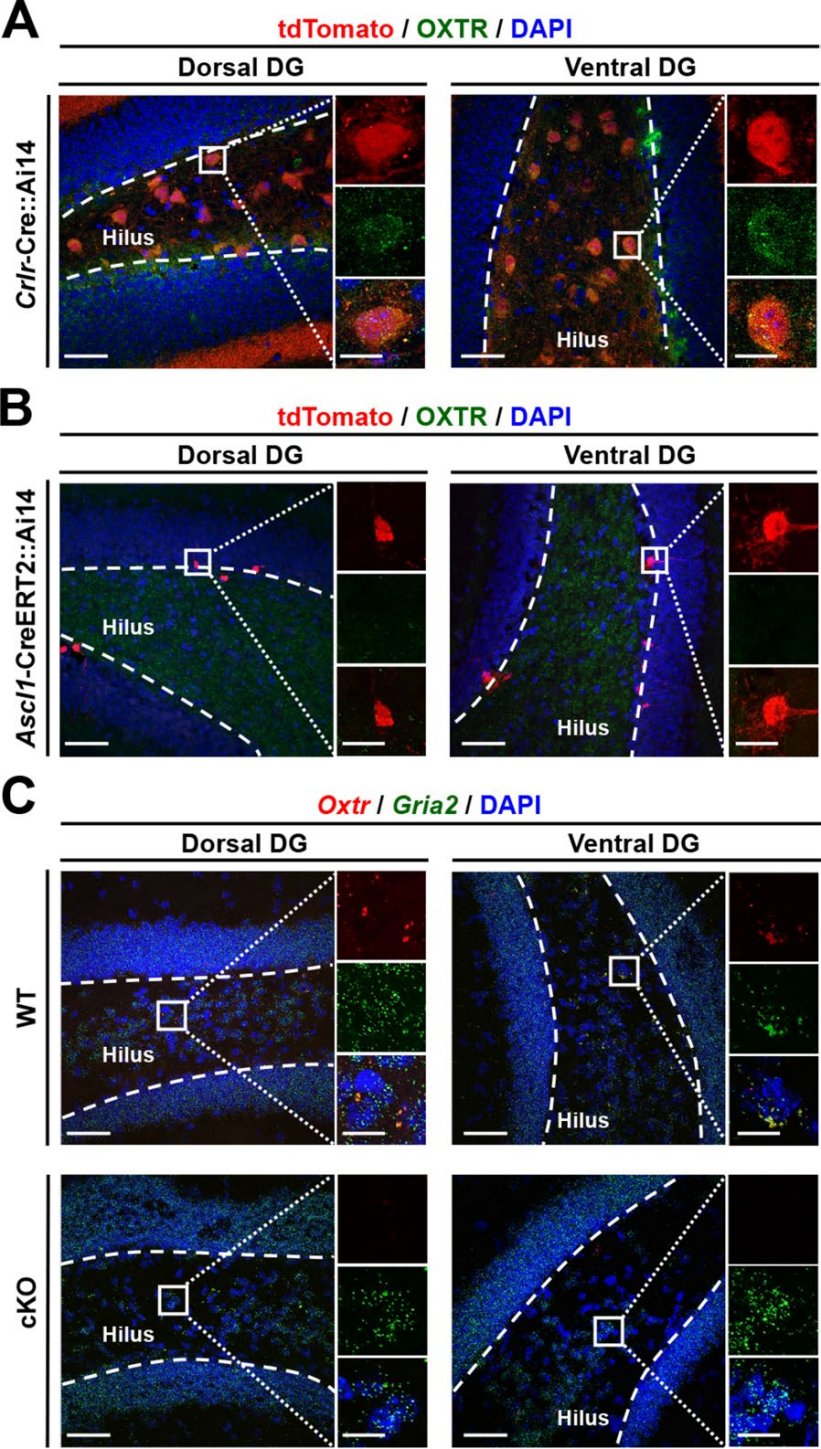
Characterization of OXTR expression on hilar MCs but not NSPCs. **A** Doubled-labeled confocal immunofluorescent image showing the colocalization of tdTomato (red) and OXTRs (green) in dorsal and ventral DG of *Crlr*-Cre::Ai14 mice. Scale bar: 50 μm. The right panels are higher-magnification views of the boxed areas in the left panel, highlighting a representative OXTR^+^tdTomato^+^ cell. Scale bar, 10 µm. Data was replicated in 4 mice. **B** Doubled-labeled confocal immunofluorescent images showing the absence of OXTRs (green) expression on tdTomato^+^ (red) cells in dorsal and ventral DG of *Ascl1*-CreERT2::Ai14 mice after 4-hydroxytamoxifen induction. Scale bar: 50 μm. The right panels are higher-magnification views of the boxed areas in the left panel. Scale bar, 10 µm. Data was replicated in 4 mice. **C** Dual-probe FISH images showing the coexpression of *Oxtr* mRNA (red) and *Gria2* mRNA (green) in dorsal and ventral DG of WT mice but not cKO mice (counterstained with DAPI, blue). Scale bar, 50 μm. The right panels are higher-magnification views of the boxed areas in the left panel. Scale bar, 10 µm. Data was replicated in 4 mice

We first performed immunohistochemistry to validate OXTR expression in the DG of adult mice. We employed genetically modified mice to conditionally express tdTomato fluorescence in MCs by crossing transgenic Ai14 reporter mice with *Crlr*-Cre mice, in which Cre expression is mainly restricted to MCs [30]. Consistent with our previous findings [22, 27], the majority of tdTomato-expressing cells were located in the hilus of dorsal and ventral DG. Double-labeling experiments revealed that tdTomato-expressing cells were also immunoreactive for OXTRs, indicating that OXTRs in the DG are mainly expressed in hilar MCs (Fig. 1A). To better verify whether OXTRs are expressed on NSPCs, we took advantage of *Ascl1*-CreERT2::Ai14 mice to label large cohorts of newly generated DGCs [31, 32]. Two weeks after 4-hydroxytamoxifen treatment, tdTomato-expressing adult-born DGCs were consistently observed in the subgranular zone (SGZ) and GC layer of the DG. However, none of the tdTomato-expressing cells were OXTR immunoreactive in dorsal or ventral DG (Fig. 1B). To gain insight into the functional role of MC OXTRs in regulating AHN, we used the Cre-loxP recombination approach to conditionally delete *Oxtr* from MCs (cKO) by crossing loxP-flanked *Oxtr* (*Oxtr*^f/f^) transgenic mice with *Crlr*-Cre mice. A dual-probe FISH revealed that the majority of *Oxtr* mRNA-positive cells were MC marker *Gria2* mRNA-expressing cells, and the numbers of *Oxtr*-positive cells in both dorsal and ventral DG of cKO mice were significantly reduced compared with those of WT (*Oxtr*^f/f^) mice (Fig. 1C), confirming the effectiveness of Cre-loxP-mediated deletion of the MC *Oxtr*. Collectively, these results demonstrate that OXTRs are selectively enriched in hilar MCs but not NSPCs that reside within the SGZ or mature GCs of the adult DG.

### MC *Oxtr* deletion impairs the proliferation, differentiation, survival, and maturation of NSPCs

**Fig. 2:**
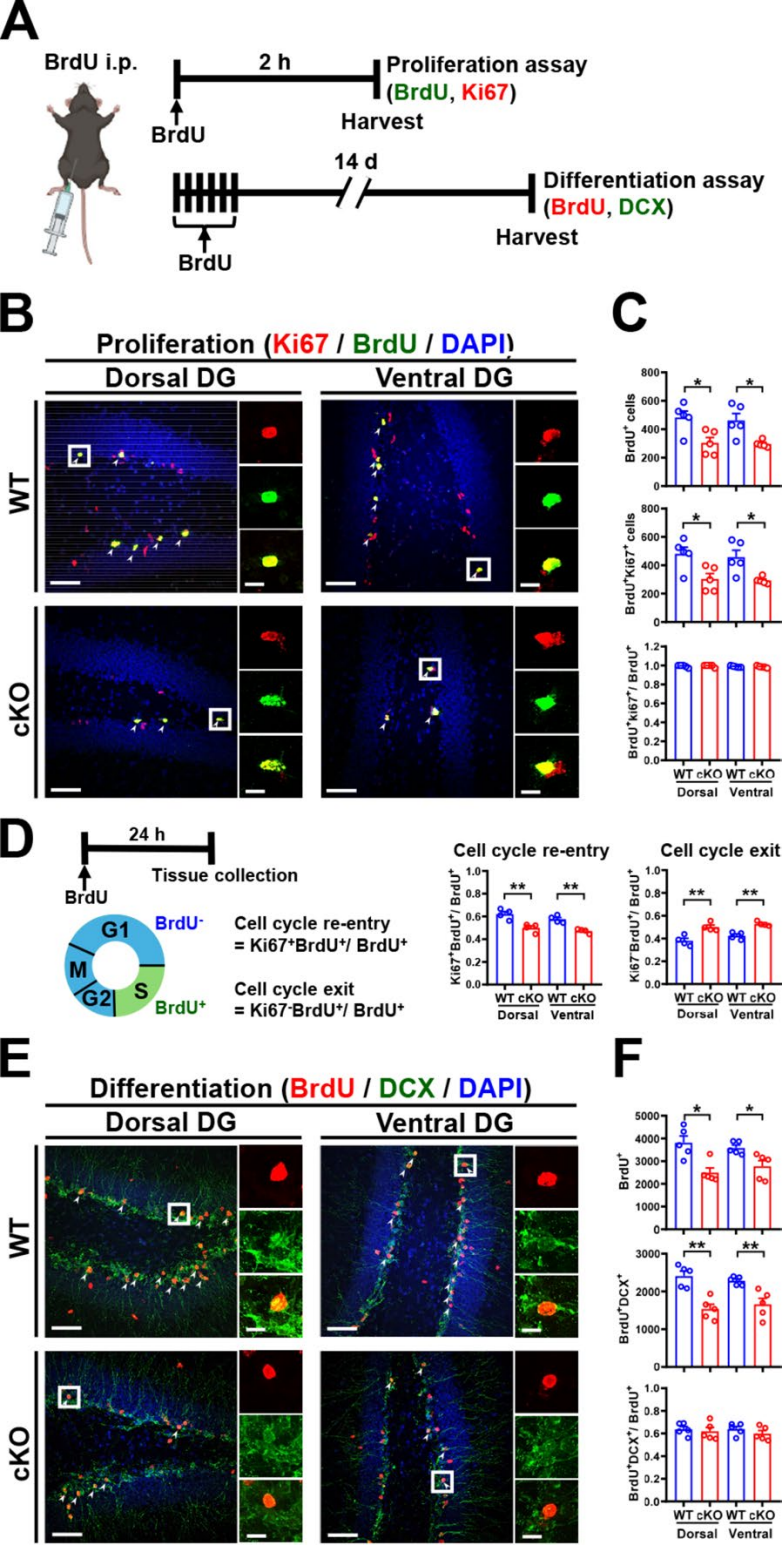
MC *Oxtr* deletion impairs the proliferation and differentiation of NSPCs. **A** Schematic representation of the experimental design. For the cell proliferation assay, mice were injected intraperitoneally with a single pulse of BrdU (50 mg/kg), and double fluorescent labeling for detecting BrdU and Ki67 was performed in both dorsal and ventral DG Sections 2 h later. For quantification of neuronal differentiation, mice were injected intraperitoneally with BrdU (50 mg/kg) 6 times at 12-h intervals, and double fluorescent labeling for detecting BrdU and DCX was performed in both dorsal and ventral DG Sects. 14 days later. **B** Representative immunofluorescence images of dorsal and ventral hippocampal DG sections from WT and cKO mice triple-stained for BrdU (green), Ki67 (red), and DAPI (blue) 2 h after BrdU injection. The white arrowhead marks Ki67^+^BrdU^+^DAPI^+^ cells. Scale bar, 50 µm. The right panels are higher-magnification views of the boxed areas in the left panel. Scale bar, 10 µm. **C** Quantification of the total number of BrdU^+^ cells (two-tailed unpaired Student's *t*-test, Dorsal: *t*_(8)_ = 3.020, *P* = 0.0166; Ventral: *t*_(8)_ = 3.303, *P* = 0.0108; *n* = 5 in each group), Ki67^+^BrdU^+^ cells (Dorsal: *t*_(8)_ = 2.908, *P* = 0.0197; Ventral: *t*_(8)_ = 3.238, *P* = 0.0119), and Ki67^+^BrdU^+^/BrdU^+^ (Dorsal: *t*_(8)_ = 0.3391, *P* = 0.7433; Ventral: *t*_(8)_ = 0.4201, *P* = 0.6855) in dorsal and ventral DG of WT and cKO mice 2 h after BrdU injection. **D** Left: schematic representation of the analysis of cell-cycle kinetics by a BrdU/Ki67 double labeling and of the index used for the cell cycle re-entry and exit. Middle: decreased proportion of cells re-entering (Ki67^+^BrdU^+^/BrdU^+^) the cell cycle in dorsal and ventral DG of cKO mice (two-tailed unpaired Student's *t*-test, Dorsal: *t*_(6)_ = 4.117, *P* = 0.0062; Ventral: *t*_(6)_ = 5.491, *P* = 0.0015; *n* = 4 in each group). Right: increased proportion of cells exiting (Ki67^−^BrdU^+^/BrdU^+^) the cell cycle in dorsal and ventral DG of cKO mice (two-tailed unpaired Student’s *t*-test, Dorsal: *t*_(6)_ = 4.117, *P* = 0.0062; Ventral: *t*_(6)_ = 5.491, *P* = 0.0015; *n* = 4 in each group). **E** Representative immunofluorescence images of dorsal and ventral hippocampal DG sections from WT and cKO mice triple-stained for BrdU (red), DCX (green), and DAPI (blue) 14 days after the last BrdU injection. The white arrowhead marks DCX^+^BrdU^+^DAPI^+^ cells. Scale bar, 50 µm. The right panels are higher-magnification views of the boxed areas in the left panel. Scale bar, 10 µm. **F** Quantification of the total number of BrdU^+^ cells (two-tailed unpaired Student's *t*-test, Dorsal: *t*_(8)_ = 3.695, *P* = 0.0061; Ventral: *t*_(8)_ = 2.89, *P* = 0.0202; *n* = 5 in each group), DCX^+^BrdU^+^ cells (Dorsal: *t*_(8)_ = 4.868, *P* = 0.0012; Ventral: *t*_(8)_ = 3.723, *P* = 0.0058), and DCX^+^BrdU^+^/BrdU^+^ (Dorsal: *t*_(8)_ = 0.4332, *P* = 0.6763; Ventral: *t*_(8)_ = 1.151, *P* = 0.2830) in dorsal and ventral DG of WT and cKO mice 14 days after the last BrdU injection. Data are presented as mean ± SEM. *P < 0.05 and **P < 0.01 as compared with WT mice

AHN is a highly dynamic process that comprises multiple stages, including cell proliferation, neuronal differentiation, survival, and maturation [33]. We then conducted a series of experiments to examine the effects of MC *Oxtr* deletion on different stages of AHN. We traced newborn cells by BrdU labeling. To determine whether MC OXTR functionally interacts with the proliferation of NSPCs, WT and cKO mice were injected with a single pulse of BrdU, and double fluorescent labeling for detection of BrdU-positive (BrdU^+^) proliferating cells (Ki67^+^) was performed in both dorsal and ventral DG Sections 2 h later (Fig. 2A–C). Stereological analysis revealed a significant reduction in the total numbers of BrdU^+^and BrdU^+^Ki67^+^ cells in cKO mice compared with WT mice in both dorsal and ventral DG (Fig. 2C). However, the proliferation rate of NSPCs, calculated as the percentage of BrdU^+^Ki67^+^ cells to total BrdU^+^ cells, was similar between WT and cKO mice (Fig. 2C). To determine whether MC *Oxtr* deletion affects cell cycle progression in NSPCs, the number of cells re-entering (BrdU^+^Ki67^+^/BrdU^+^) and exiting the cell cycle (BrdU^+^Ki67^−^/BrdU^+^) was calculated (Fig. 2D). Cell cycle progression was assessed in both dorsal and ventral DG Sects. 24 h following BrdU injection. We found that the proportion of cells re-entering the cell cycle was significantly reduced in cKO mice compared with WT mice. By contrast, a significantly higher proportion of cells exited the cell cycle in cKO mice compared with WT mice (Fig. 2D). To determine whether MC *Oxtr* deletion may affect the neuronal differentiation of NSPCs, WT and cKO mice were subjected to multiple BrdU injections (Fig. 2A), and double fluorescent labeling for detection of BrdU and immature neurons (DCX^+^) was performed in both dorsal and ventral DG Sects. 14 days later (Fig. 2E, F). Quantitative analysis of neuronal differentiation revealed a significant decrease in the total numbers of BrdU^+^, DCX^+^, and BrdU^+^DCX^+^ cells in cKO mice 14 days after BrdU injections compared with WT mice in both dorsal and ventral DG (Fig. 2F). However, analysis of the percentage of BrdU^+^DCX^+^ cells to total BrdU^+^ cells revealed no significant difference between WT and cKO mice, indicating that the neuronal differentiation rate was unaffected by MC *Oxtr* deletion.

**Fig. 3:**
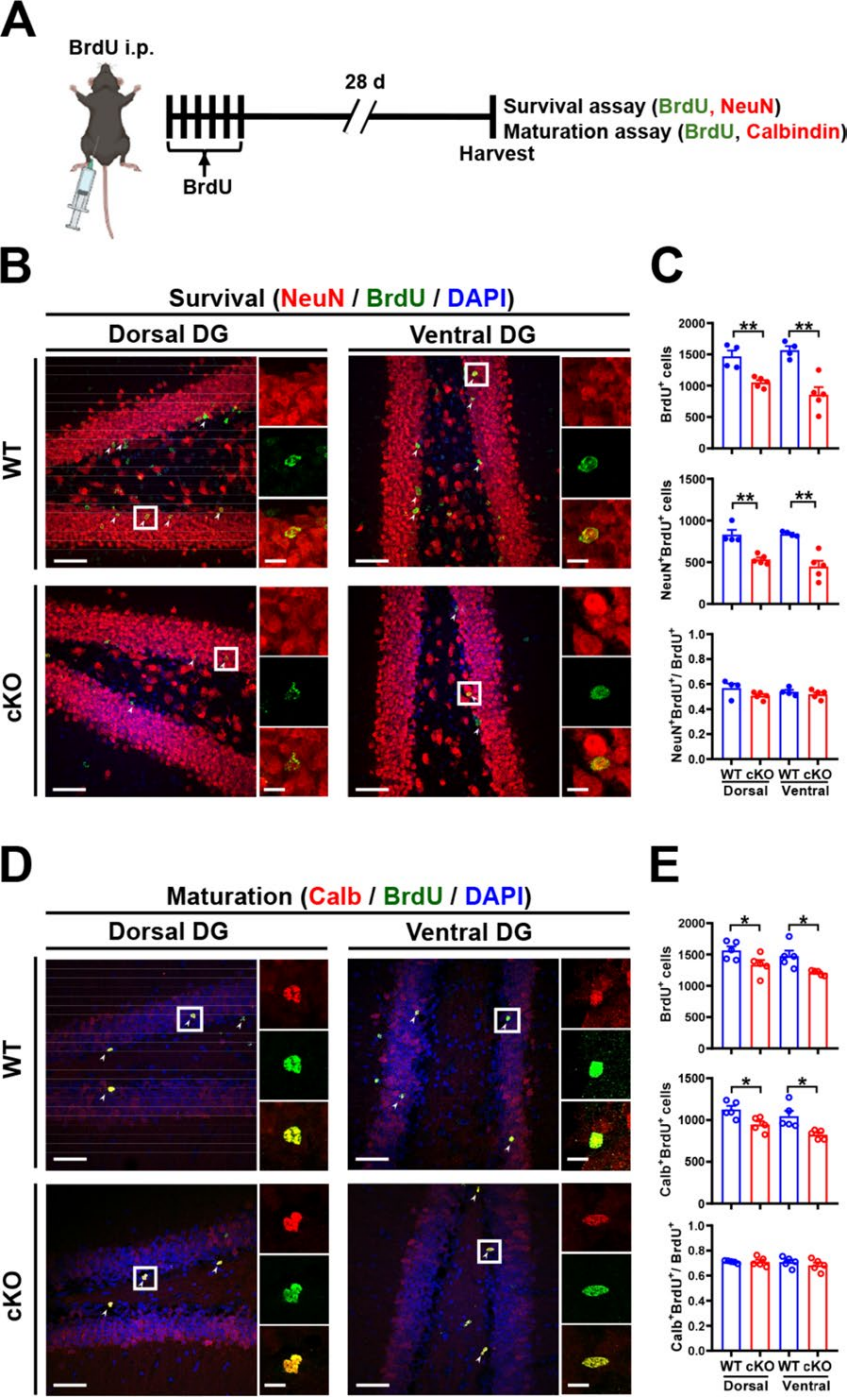
MC *Oxtr* deletion impairs the survival and maturation of NSPCs. **A** Schematic representation of the experimental design. For quantification of neuronal survival, mice were injected intraperitoneally with BrdU (50 mg/kg) 6 times at 12-h intervals, and double fluorescent labeling for detection of BrdU and NeuN was performed in both dorsal and ventral DG Sects. 28 days later. For quantification of neuronal maturation, double fluorescent labeling for detection of BrdU and calbindin (Calb) was performed in both dorsal and ventral DG Sects. 28 days later. **B** Representative immunofluorescence images of dorsal and ventral hippocampal DG sections from WT and cKO mice triple-stained for NeuN (red), BrdU (green), and DAPI (blue) 28 days after the last BrdU injection. The white arrowhead marks NeuN^+^BrdU^+^DAPI^+^ cells. Scale bar, 50 µm. The right panels are higher-magnification views of the boxed areas in the left panel. Scale bar, 10 µm. **C** Quantification of the total number of BrdU^+^ cells (two-tailed unpaired Student's *t*-test, Dorsal: *t*_(7)_ = 4.496, *P* = 0.0028; Ventral: *t*_(7)_ = 4.838, *P* = 0.0019; *n*_wt_ = 4 and *n*_cKO_ = 5), NeuN^+^BrdU^+^ cells (Dorsal: *t*_(7)_ = 5.168, *P* = 0.0013; Ventral: *t*_(7)_ = 4.933, *P* = 0.0017), and NeuN^+^BrdU^+^/BrdU^+^ (Dorsal: *t*_(7)_ = 1.815, *P* = 0.1124; Ventral: *t*_(7)_ = 0.8719, *P* = 0.4122) in dorsal and ventral DG of WT and cKO mice 28 days after the last BrdU injection. **D** Representative immunofluorescence images of dorsal and ventral hippocampal DG sections from WT and cKO mice triple-stained for Calb (red), BrdU (green), and DAPI (blue) 28 days after the last BrdU injection. The white arrowhead marks Calb^+^BrdU^+^DAPI^+^ cells. Scale bar, 50 µm. The right panels are higher-magnification views of the boxed areas in the left panel. Scale bar, 10 µm. **E** Quantification of the total number of BrdU^+^ cells (two-tailed unpaired Student's *t*-test, Dorsal: *t*_(8)_ = 2.325, *P* = 0.0485; Ventral: *t*_(8)_ = 3.055, *P* = 0.0157; *n* = 5 in each group), Calb^+^BrdU^+^ cells (Dorsal: *t*_(8)_ = 3.103, *P* = 0.0146; Ventral: *t*_(8)_ = 3.178, *P* = 0.013), and Calb^+^BrdU^+^/BrdU^+^ (Dorsal: *t*_(8)_ = 0.02918, *P* = 0.9774; Ventral: *t*_(8)_ = 0.8737, *P* = 0.4077) in dorsal and ventral DG of WT and cKO mice 28 days after the last BrdU injection. Data are presented as mean ± SEM. *P < 0.05 and **P < 0.01 as compared with WT mice

To assess whether MC *Oxtr* deletion may affect the survival of newly generated DGCs, WT and cKO mice were subjected to multiple BrdU injections (Fig. 3A), and double fluorescent labeling for detection of BrdU and mature neurons (NeuN^+^) was performed in both dorsal and ventral DG Sects. 28 days later. Quantitative analysis of the neuronal survival revealed a significant decrease in the total numbers of BrdU^+^, NeuN^+^, and BrdU^+^NeuN^+^ cells in cKO mice 28 days after BrdU injection compared with WT mice in both dorsal and ventral DG (Fig. 3B, C). However, analysis of the percentage of BrdU^+^NeuN^+^ cells to total BrdU^+^ cells revealed no significant differences between WT and cKO mice, indicating that the neuronal survival rate is unaffected by MC *Oxtr* deletion. We also used calbindin (Calb) antibody, a marker of mature GCs [34], co-immunostained with BrdU on the brain sections to measure newborn neuron maturation 28 days after BrdU injection (Fig. 3D). Noticeably, cKO mice showed a significant reduction in the total number of BrdU^+^Calb^+^ cells compared with WT mice in both dorsal and ventral DG (Fig. 3D, E). However, analysis of the percentage of BrdU^+^Calb^+^ cells to total BrdU^+^ cells revealed no difference between WT and cKO mice (Fig. 3E). These results indicated that MC OXTRs play an important role in regulating the number, but not the rate, of proliferation, differentiation, survival, and maturation of newly generated DGCs.

### MC *Oxtr* deletion reduces NSPC pools

**Fig. 4:**
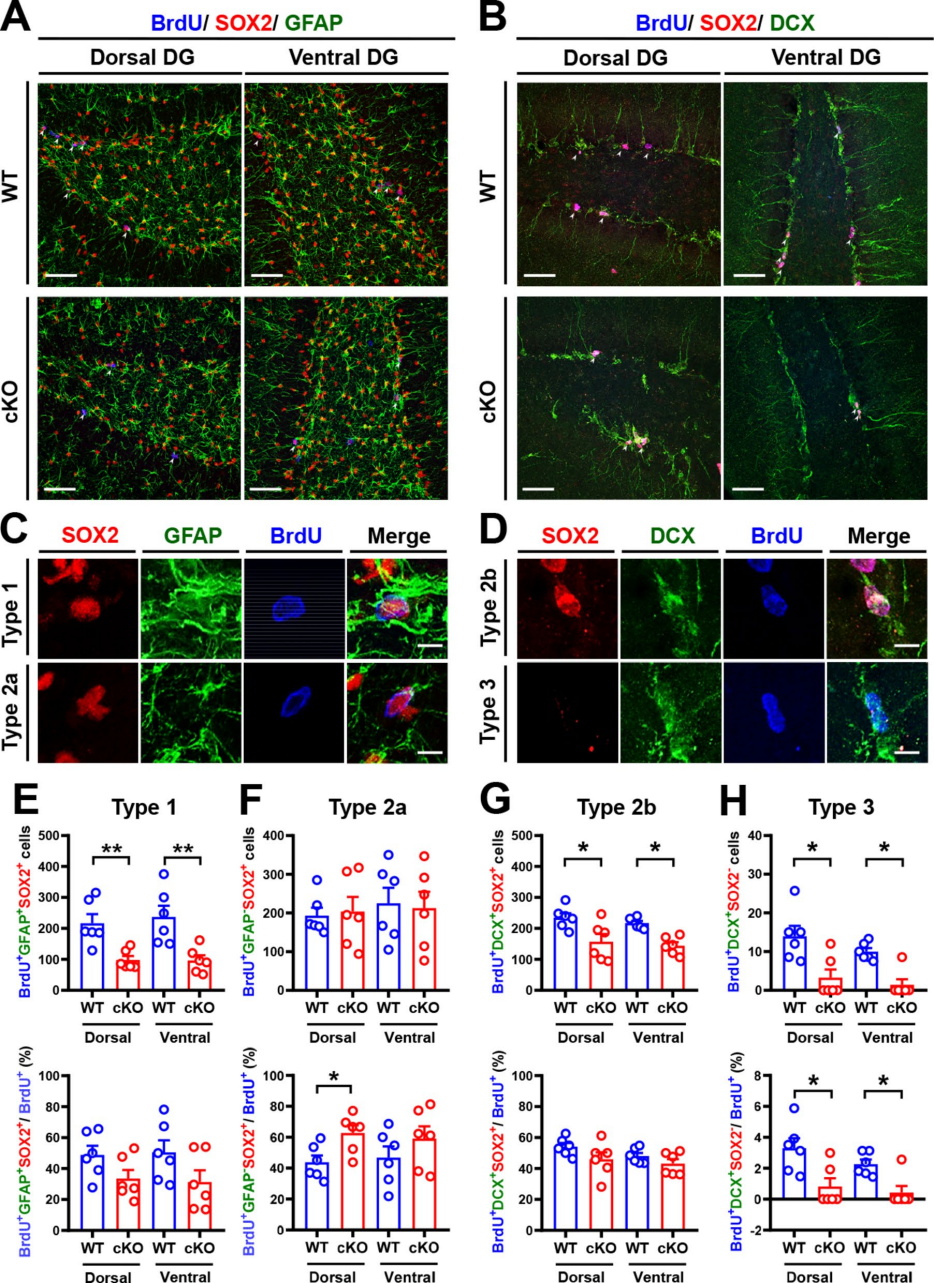
Effects of MC *Oxtr* deletion on individual NSPC populations.** A** Representative immunofluorescence images of dorsal and ventral hippocampal DG sections from WT and cKO mice triple-stained for BrdU (blue), SOX2 (red), and GFAP (green) 2 h after BrdU (50 mg/kg) injection. The white arrowhead marks BrdU^+^SOX2^+^GFAP^+^ cells. Scale bar: 100 μm. **B** Representative immunofluorescence images of dorsal and ventral hippocampal DG sections from WT and cKO mice triple-stained for BrdU (blue), SOX2 (red), and DCX (green) 2 h after BrdU injection. The white arrowhead marks BrdU^+^SOX2^+^DCX^+^ cells. Scale bar: 100 μm. **C** Representative immunofluorescence images of type 1 (BrdU^+^GFAP^+^SOX2^+^) radial glia-like stem cells and type 2a (BrdU^+^GFAP^−^SOX2^+^) transient amplifying progenitor cells. Scale bar: 5 μm. **D** Representative immunofluorescence images of type 2b (BrdU^+^SOX2^+^DCX^+^) transient amplifying progenitor cells and type 3 (BrdU^+^SOX2^−^DCX^+^) neuroblasts. Scale bar: 5 μm. **E** Upper, quantification of the total number of type 1 (BrdU^+^SOX2^+^GFAP^+^) NSPCs in dorsal and ventral DG of WT and cKO mice (two-tailed unpaired Student's *t*-test, Dorsal: *t*_(10)_ = 3.726, *P* = 0.0039; Ventral: *t*_(10)_ = 3.518, *P* = 0.0056; *n* = 6 in each group). Bottom: quantification of the percentage of BrdU^+^SOX2^+^GFAP^+^/BrdU^+^ in dorsal and ventral DG of WT and cKO mice (Dorsal: *t*_(10)_ = 1.877, *P* = 0.0899; Ventral: *t*_(10)_ = 1.765, *P* = 0.1081). **F** Upper, quantification of the total number of type 2a (BrdU^+^SOX2^+^GFAP^−^) NSPCs in dorsal and ventral DG of WT and cKO mice (two-tailed unpaired Student's *t*-test, Dorsal: *t*_(10)_ = 0.2436, *P* = 0.8124; Ventral: *t*_(10)_ = 0.2154, *P* = 0.8338; *n* = 6 in each group). Bottom: quantification of the percentage of BrdU^+^SOX2^+^GFAP^−^/BrdU^+^ in dorsal and ventral DG of WT and cKO mice (Dorsal: *t*_(10)_ = 2.815, *P* = 0.0183; Ventral: *t*_(10)_ = 1.147, *P* = 0.2782). **G** Upper, quantification of the total number of type 2b (BrdU^+^SOX2^+^DCX^+^) NSPCs in dorsal and ventral DG of WT and cKO mice (two-tailed unpaired Student's *t*-test, Dorsal: *t*_(10)_ = 2.687, *P* = 0.0228; Ventral: *t*_(10)_ = 5.404, *P* = 0.0003; *n* = 6 in each group). Bottom: quantification of the percentage of BrdU^+^SOX2^+^DCX^+^/BrdU^+^ in dorsal and ventral DG of WT and cKO mice (Dorsal: *t*_(10)_ = 1.604, *P* = 0.1398; Ventral: *t*_(10)_ = 1.386, *P* = 0.1960). **H** Upper, quantification of the total number of type 3 (BrdU^+^SOX2^−^DCX^+^) NSPCs in dorsal and ventral DG of WT and cKO mice (two-tailed Mann–Whitney *U* test, Dorsal: *P* = 0.0152; Ventral: *P* = 0.0087; *n* = 6 in each group). Bottom: quantification of the percentage of BrdU^+^SOX2^−^DCX^+^/BrdU^+^ in dorsal and ventral DG of WT and cKO mice (two-tailed Mann–Whitney *U* test, Dorsal: *P* = 0.0195; Ventral: *P* = 0.0216). Data are presented as mean ± SEM. *P < 0.05 and **P < 0.01 as compared with WT mice

Adult hippocampal NSPC pools consist of three major cell subtypes: radial glia-like precursor cells (type 1), transient-amplifying progenitors (types 2a and 2b), and neuroblasts (type 3) [35]. We next asked whether MC *Oxtr* deletion affects the preservation of NSPC populations. To examine this, we analyzed the number of cells positive for GFAP and SOX2 2 h after BrdU injection (Fig. 4A–D). We found a significant reduction in the numbers of type 1 (BrdU^+^GFAP^+^SOX2^+^), type 2b (BrdU^+^SOX2^+^DCX^+^), and type 3 (BrdU^+^SOX2^−^DCX^+^) cells in both dorsal and ventral DG of cKO mice compared with WT mice (Fig. 4E, G, H), but the number of type 2a (BrdU^+^GFAP^−^SOX2^+^) cells remained unaltered in cKO mice (Fig. 4F). There were no significant differences in the percentages of type 1 and type 2b cells to total BrdU^+^ cells in both dorsal and ventral DG of cKO mice compared with WT mice (Fig. 4E, G). However, a significant reduction in the percentage of type 3 neuroblasts (BrdU^+^SOX2^−^DCX^+^) to total BrdU^+^ cells in both dorsal and ventral DG of cKO mice compared with WT mice (Fig. 4H). These results suggest that MC *Oxtr* deletion reduces NSPC pools.

**Fig. 5:**
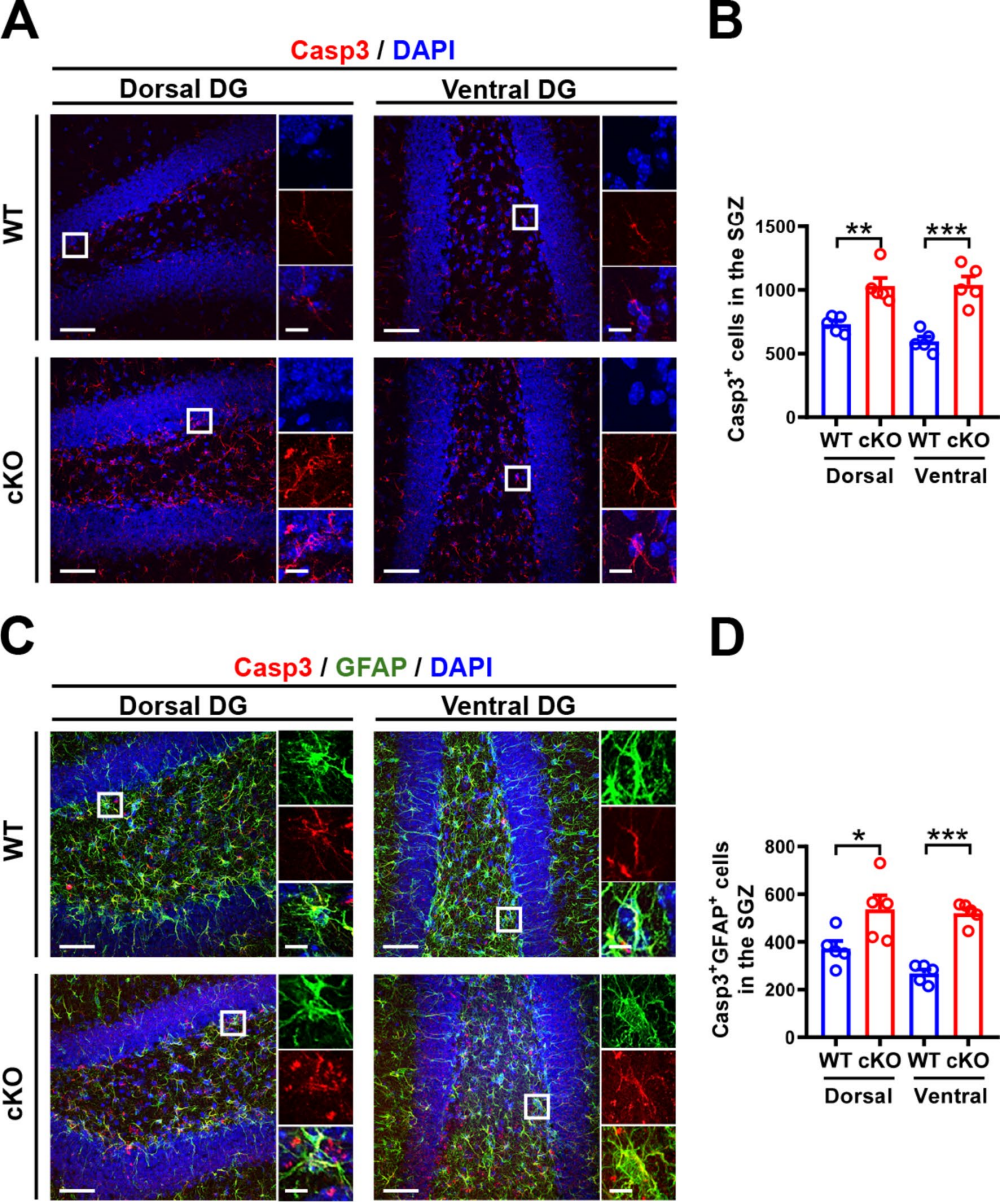
MC *Oxtr* deletion increases cell death in the SGZ.** A** Representative immunofluorescence images of dorsal and ventral hippocampal DG sections from WT and cKO mice stained for activated caspase-3 (Casp3). Scale bar, 50 μm. The right panels are higher-magnification views of the boxed areas in the left panel. Scale bar, 10 µm. **B** Quantification of the total number of Casp3^+^ cells in the SGZ of WT and cKO mice (two-tailed unpaired Student's *t*-test, Dorsal: *t*_(8)_ = 4.263, *P* = 0.0027; Ventral: *t*_(8)_ = 5.849, *P* = 0.0004; *n* = 5 in each group). **C** Representative immunofluorescence images of dorsal and ventral hippocampal DG sections from WT and cKO mice double-stained for GFAP (blue) and Casp3 (red). Scale bar, 50 μm. The right panels are higher-magnification views of the boxed areas in the left panel, highlighting representative GFAP^+^Casp3^+^ cells. Scale bar, 10 µm. **D** Quantification of the total number of GFAP^+^Casp3^+^ cells in the SGZ of WT and cKO mice (two-tailed unpaired Student's *t*-test, Dorsal: *t*_(8)_ = 2.447, *P* = 0.0401; Ventral: *t*_(8)_ = 9.524, *P* < 0.0001; *n* = 5 in each group). Data are presented as mean ± SEM. *P < 0.05, **P < 0.01, and *** *P* < 0.001 as compared with WT mice

We further investigated whether deletion of MC Oxtr reduces the NSPC pool by increasing cell death, as indicated by staining for activated caspase-3, a marker of apoptosis [36, 37]. Quantification showed a significant increase in the number of active caspase-3-positive (Casp3^+^) cells in the DG of cKO mice compared with WT mice, indicating increased cell death (Fig. 5A, B). A notable increase in the number of type 1 NSPCs expressing Casp3^+^ was observed in the DG of cKO mice compared to WT mice (Fig. 5C, D), which might contribute to the reduction in NSPC pools.

### MC *Oxtr* deletion retards the dendritic development of newly generated DGCs

**Fig. 6:**
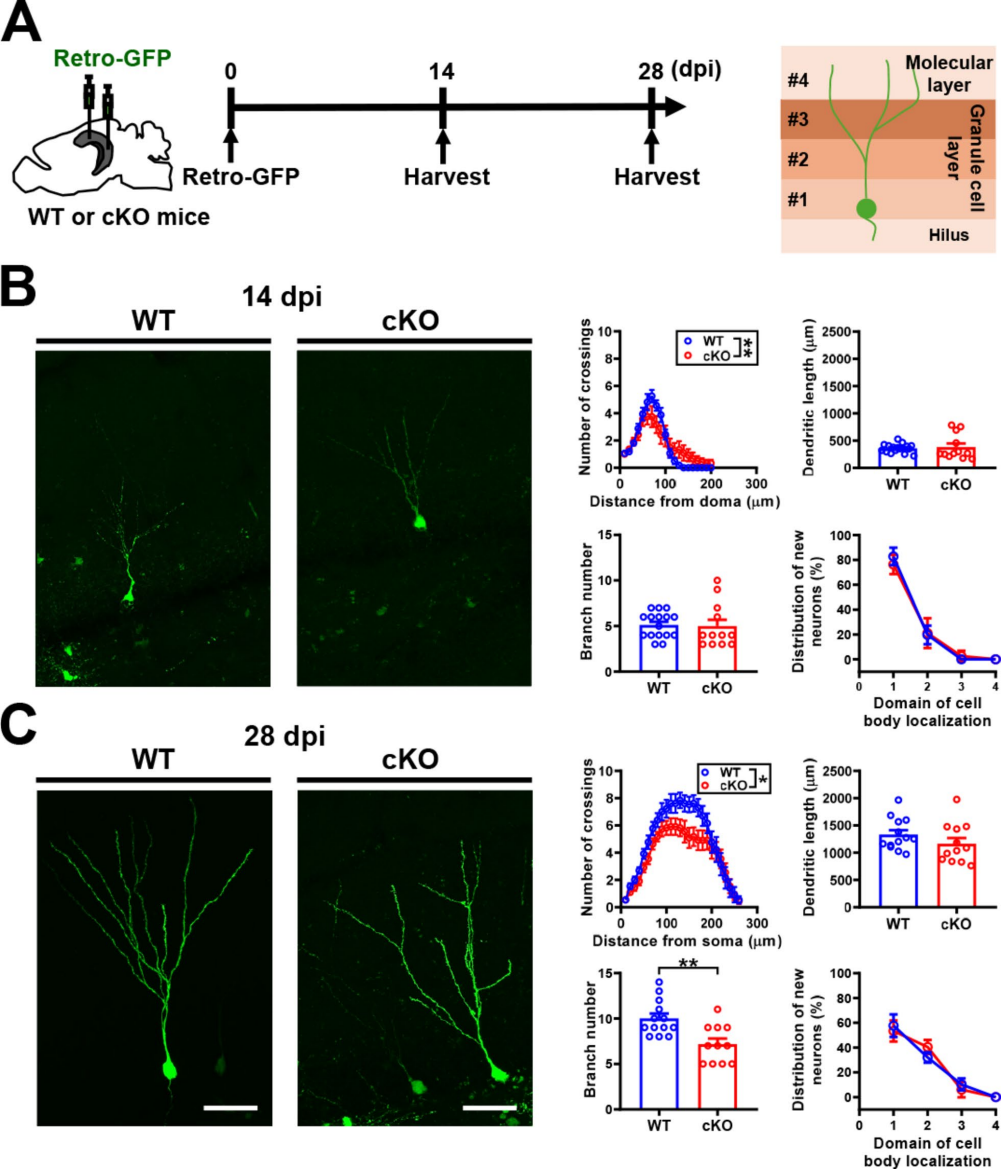
MC *Oxtr* deletion impairs the dendritic development of newly generated DGCs. **A** Left: schematic representation of the experimental design. Retroviral expression vector encoding GFP (Retro-GFP) was stereotaxically microinjected into the DG hilus of WT and cKO mice. Sholl analysis of dendritic complexity in GFP^+^ DGCs was performed at 14 and 28 dpi. Right: Schematic diagram of the DG region divided into 4 domains.** B** Left: representative images of GFP^+^ DGCs from WT and cKO mice at 14 dpi. Scale bar, 50 µm. Right: summary bar graphs depicting the number of crossings (two-way RM ANOVA, Distance: *F*_(19,494)_ = 39.2, *P* < 0.0001; Genotype: *F*_(1,26)_ = 0.0057, *P* = 0.9399; Interaction: *F*_(19,494)_ = 2.95, *P* < 0.0001; *n* = 4 in each group), total dendritic length (two-tailed unpaired Student's *t*-test, *t*_(26)_ = 0.3676, *P* = 0.7162), branching number (two-tailed unpaired Student's *t*-test, *t*_(26)_ = 0.1706, *P* = 0.8659), and distribution of GFP^+^ DGCs (Two-way RM ANOVA, Distance: *F*_(3,12)_ = 153.7, *P* < 0.0001; Genotype: *F*_(1,4)_ = 1.0, *P* = 0.3739; Interaction: *F*_(3,12)_ = 0.4583, *P* < 0.7165) from WT and cKO mice at 14 dpi. **C** Left: representative images of GFP^+^ DGCs from WT and cKO mice at 28 dpi. Scale bar, 50 µm. Right: summary bar graphs depicting the number of crossings (two-way RM ANOVA, Distance: *F*_(25,550)_ = 41.04, *P* < 0.0001; Genotype: *F*_(1,22)_ = 4.897, *P* = 0.0376; Interaction: *F*_(25,550)_ = 1.621, *P* = 0.0298; *n* = 4 in each group), total dendritic length (two-tailed unpaired Student's *t*-test, *t*_(23)_ = 1.290, *P* = 0.21), branching number (two-tailed unpaired Student's *t*-test, *t*_(22)_ = 3.408, *P* = 0.0025), and distribution of GFP^+^ DGCs (two-way RM ANOVA, Distance: *F*_(1.293, 5.173)_ = 87.7, *P* = 0.0002; Genotype: *F*_(1,4)_ = 0.071, *P* = 0.80; Interaction: *F*_(3,12)_ = 1.18, *P* = 0.3583) from WT and cKO mice at 28 dpi. Data are presented as mean ± SEM. *P < 0.05 and **P < 0.01 as compared with WT mice

We next determined whether MC *Oxtr* deletion affects the morphological maturation, migration, and cell positioning of newly generated DGCs using a retrovirus-mediated birthdating and cell-labeling strategy [22, 29]. Engineered retroviruses that express GFP were stereotaxically microinjected into the DG hilus of WT and cKO mice. Sholl analysis of dendritic complexity in GFP^+^ DGCs was performed at 14 and 28 dpi (Fig. 6A). Using confocal microscopy to reconstruct the dendritic arborization of GFP^+^ DGCs, we found that newly generated DGCs from cKO mice exhibited less elaborated dendritic arborization than those from WT mice at 14 dpi (Fig. 6B). No significant differences in total dendritic length, branch number, and positioning of GFP^+^ DGCs were observed between WT and cKO mice at 14 dpi. At 28 dpi, we observed significant deficits in dendritic complexity and branch number between WT and cKO mice (Fig. 6C). The total dendritic length and distribution of GFP^+^ DGCs, however, were not significantly different between WT and cKO mice. These results indicated that MC *Oxtr* deletion impairs the maturation process of newly generated DGCs.

### MC *Oxtr* deletion impairs contextual fear discrimination

**Fig. 7:**
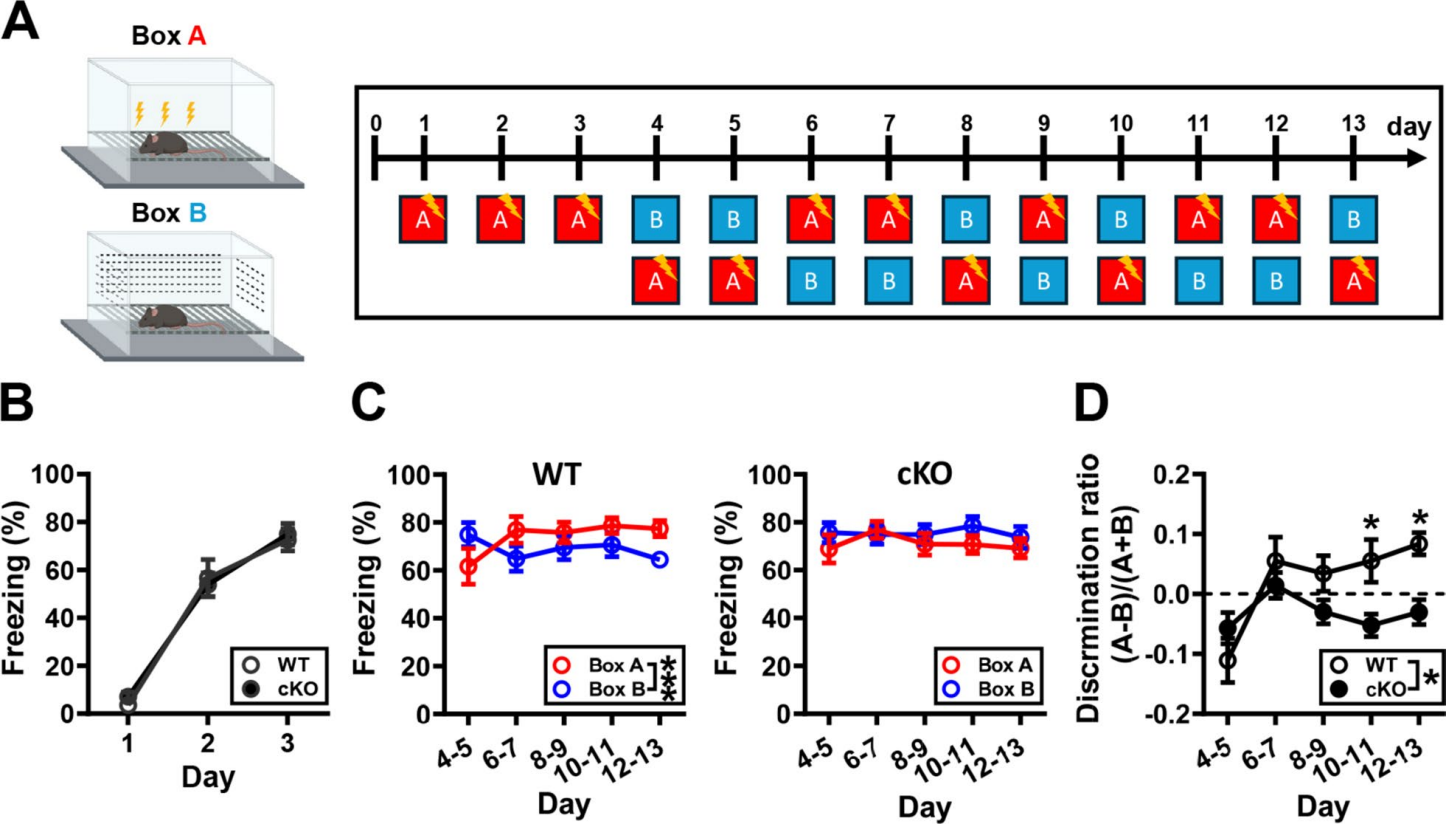
MC *Oxtr* deletion impairs contextual fear discrimination. **A** Left, schematic of context A and B used in contextual fear discrimination (see Materials and Methods). Right, experimental procedure for the contextual fear discrimination learning task. During the first 3 days, mice were placed only in context A and received a single footshock 3 min later. From day 4 to day 13, mice were placed in either context A or B to measure freezing levels in counterbalanced order. **B** Summary graph showing the percentage of freezing during first 3 days in Context A (conditioning context) (two-way RM ANOVA, Group: *F*_(1,20)_ = 0.04015, *P* = 0.8432; Time: *F*_(2,40)_ = 203.3, *P* < 0.0001; Interaction: *F*_(2,40)_ = 0.4463, *P* = 0.6431; *n* = 11 in each group). **C** Left: summary graph showing the percentage of freezing from day 4 to day 13 in Context A and B (unconditioning context) of WT mice (two-way RM ANOVA, Context: *F*_(1,18)_ = 0.8828, *P* = 0.3599; Time: *F*_(3.217,57.91)_ = 1.050, *P* = 0.3804; Interaction: *F*_(4,72)_ = 5.365, *P* = 0.0008). Right: summary graph showing the percentage of freezing from day 4 to day 13 in Context A and B of cKO mice (two-way RM ANOVA, Context: *F*_(1,18)_ = 0.6804, *P* = 0.4202; Time: *F*_(3.414,61.45)_ = 0.9508, *P* = 0.4306; Interaction: *F*_(4,72)_ = 1.066, *P* = 0.3796). **D** Summary graph showing the contextual discrimination ratio from day 4 to day 13 in WT and cKO mice (two-way RM ANOVA, Group: *F*_(1,19)_ = 5.635, *P* = 0.0283; Time: *F*_(4,76)_ = 6.479, *P* = 0.0002; Interaction: *F*_(4,76)_ = 3.346, *P* = 0.0141; Sidak’s post hoc multiple comparisons test, day 10–11: *P* = 0.044, day 12–13: *P* = 0.0283). Data are presented as mean ± SEM. *P < 0.05 and ***P < 0.001 as compared with WT mice

AHN is functionally connected to modulating hippocampal memory interference, particularly in processes like pattern separation [38, 39]. To further understand the functional impact of MC *Oxtr* deletion-induced impairment of AHN, we used a contextual fear discrimination learning task to assess hippocampal memory interference. In this test, we subjected WT and cKO mice to contextual fear conditioning using a pair of similar contexts, A and B. As shown in Fig. 7A, during the first 3 days, mice were only placed in context A and received a single footshock after 180 s. There was no significant difference in the percentage of freezing during the learning phase between WT and cKO mice (Fig. 7B). From day 4 to day 13, mice were placed in either context A, which is associated with a single footshock, or context B, which is not (Fig. 7C, D). Initially, both WT and cKO mice were unable to discriminate between contexts, resulting in similar freezing levels in both contexts (days 4–5). As the experiment progressed, WT mice gained the ability to effectively discriminate context B from context A, while cKO mice did not, and the discrimination ratio increased (Fig. 7C). On days 10–11 and 12–13, cKO mice showed significant difficulties in acquiring discrimination abilities and exhibited a lower discrimination ratio than WT mice (Fig. 7D), suggesting that MC *Oxtr* deletion leads to impaired context discrimination during fear conditioning.

## Discussion

OXTRs are expressed throughout the brain but have a very limited distribution in the hippocampus, particularly in the pyramidal neurons of CA2/CA3a and hilar MCs [22, 24, 25, 40]. Our previous study reported that conditional deletion of *Oxtr* in hippocampal excitatory neurons impairs the survival and maturation of newly generated DGCs [22]. While our previous study highlighted a non-cell-autonomous role for OXT in promoting AHN via OXTRs expressed in hippocampal CA3 pyramidal neurons, our genetic strategy using the Cre-loxP recombination system did not rule out the possibility that other OXTR-expressing cells, such as MCs, may also contribute to regulating AHN. Here, we aimed to investigate the role of MC OXTR signaling in regulating AHN and contextual discrimination abilities. In addition to confirming that OXTRs are highly abundant in MCs [22, 26], our study provides the first evidence that deletion of *Oxtr* in MCs decreases the number, but not the rate, of proliferation, differentiation, survival, and maturation of NSPCs, and retards the dendritic development of newly generated DGCs. Additionally, we demonstrate that MC *oxtr* deletion reduces NSPC pools. Overall, we describe a novel mechanism through which hilar MCs act as critical niche components that dynamically regulate AHN and associated brain functions via OXTR signaling.

A significant challenge in physiological studies of OXTRs is the limited antibody specificity, which hinders the accurate detection of cell-type expression through immunohistochemistry [41]. Recently developed novel antibodies targeting OXTRs have effectively overcome this limitation [42, 43]. Consistent with previous studies with transgenic *Oxtr*^Venus−Neo/+^ reporter mice that encode a fluorescent reporter gene under the OXTR promoter [22, 26], we find that OXTRs are abundantly expressed in hilar MCs by immunofluorescence staining using an anti-OXTR antibody in naïve mice. Using the classical BrdU pulse-labeling strategy, our previous work reported that OXTRs are not expressed in NSPCs in the SGZ or mature GCs of the DG [22]. Given that BrdU only tracks dividing NSPCs during the S phase of the cell cycle, genetically engineered mice have become powerful tools for accurately labeling entire cohorts of newly generated DGCs [44, 45]. Using these advanced genetic tools, we now present additional evidence demonstrating that tamoxifen induction in Ascl1-CreERT2::Ai14 mice labeled a cohort of newly generated DGCs that lacked OXTR immunoreactivity. These findings clearly indicate that OXTR signaling may function non-cell autonomously to regulate AHN. Although OXTRs are expressed in hilar MCs, we found a small subset of cells at the border of the GC layer that also express OXTRs (Fig. 1A) in both dorsal and ventral DG. Because our previous findings demonstrated that a small subset of hilar GABAergic interneurons within the SGZ also express OXTRs [22], these OXTR-immunoreactive cells could be GABAergic interneurons.

The process of AHN starts with the activation of quiescent NSPCs, followed by the division of intermediate progenitors and neuroblasts, their differentiation, and the integration of newborn neurons into pre-existing neural circuits [1–3]. Our previous work indicated that calcium/calmodulin-dependent protein kinase II α (CaMKIIα)-Cre-OXTR knockout impaired the survival and maturation of newly generated DGCs [22]. We have extended these findings by underscoring the importance of MC OXTRs in regulating the proliferation, differentiation, survival, and maturation of newly generated DGCs. How does genetic deletion of *oxtr* in hilar MCs lead to dysregulation of AHN? The most parsimonious explanation is that MC *Oxtr* deletion alters local neural circuit activity in the DG neurogenic niche, thereby impairing AHN. This builds on previous studies showing that MCs differentially regulate local and distant GC activity via monosynaptic excitatory connections with GCs or indirectly through GABAergic interneurons [13–15]. Additionally, MC *Oxtr* deletion was previously found to result in an increase in the spontaneous firing rate of GCs, the synaptic excitation-inhibition ratio in the MC-to-GC circuit, and GC firing when temporally linked with inputs from the lateral perforant path [26]. Heightened excitability of mature GCs may influence AHN at distinct stages by secreting growth factors or modulating morphogen signaling [19]. Given that MCs provide glutamatergic inputs onto newly generated DGCs [16–18], it is also possible that altered glutamatergic transmission in the circuit from MCs to newly generated DGCs may play a role in impaired AHN by MC *Oxtr* deletion. Indeed, previous studies have demonstrated that glutamatergic signaling via *N*-methyl-D-aspartate receptors promotes the survival and integration of newly generated DGCs [46, 47]. By using a retrovirus-mediated birthdating and cell labeling approach, we recognized that MC *oxtr* deletion retards the dendritic development of newly generated DGCs. Our findings reinforce a previous study demonstrating that MC inputs onto GCs are crucial for the maturation and integration of newborn DGCs [48].

Previous studies have shown that *Crlr*-Cre mice exhibit specific Cre recombinase expression in the DG hilus and in some nearby CA3c pyramidal cells and cerebellar Purkinje cells [26, 30, 49]. Thus, off-target Cre-mediated recombination of *Crlr*-Cre in the hippocampus may be a concern. Based on OXTR-Venus knock-in mice, it is well established that OXTRs are prominently expressed in hippocampal CA2 and CA3a, as well as in hilar MCs [22, 25, 26]. Although *Crlr*-Cre mice express Cre recombinase in CA3c pyramidal cells, these cells do not express OXTRs. In addition, our previous results, using dual-probe FISH analysis, have demonstrated that *Oxtr*-positive cells remain detectable throughout the CA2 and CA3a pyramidal cell layer of both the dorsal and ventral hippocampus in cKO mice [26]. These observations may exclude the off-target effect of Cre-mediated recombination by using *Crlr*-Cre to conditionally delete *Oxtr* from MCs of the hippocampus.

Pattern separation is a network mechanism that enables distinct, non-overlapping discrimination among similar representations [50, 51]. The hippocampal DG is considered a crucial region for pattern separation due to its sparse activity pattern [52, 53]. As newly generated DGCs can modify the activity of the entire DG, it is conceivable that AHN contributes to the pattern separation function of the DG. In addition, immature DGCs have sparse connections with incoming fibers, which may help form distinct representations of similar events, thereby preventing memory interference and enhancing the discrimination of overlapping stimuli [54]. Consistent with previous studies showing that enhancing AHN promotes contextual fear discrimination [38, 39], our results indicate that cKO mice showed impaired AHN and performed poorly in contextual fear discrimination. The current study is the first to show a clear correlation between MC *Oxtr* deletion-mediated decrease in AHN and impaired context discrimination abilities in mice. Given that MC inhibition has been linked to contextual discrimination deficit by increasing the active GC populations in response to a contextual stimulus [55], we cannot exclude the possibility that the increased mature DGC activity following MC *oxtr* deletion may also be linked to the observed contextual discrimination deficit. While anxiety often causes overgeneralization in contexts, it is worth noting that cKO mice exhibited behaviors in the open-field test similar to those of WT mice [26], suggesting that the observed contextual discrimination deficit is not associated with an alteration in anxiety state.

Previous studies have demonstrated that differential activation of MCs can regulate the balance between quiescence and activation of NSPCs through a dynamic interplay of direct and indirect pathways [11]. Consistent with this work, we observed a significant reduction in NSPC pools following MC *Oxtr* deletion. Additionally, MC *Oxtr* deletion reduced cell cycle re-entry and promoted cell cycle exit and NSPC death. Regarding the reduction in NSPC pools, it is tempting to speculate that our knockout approach might trigger mechanisms to disrupt NSPC maintenance under normal conditions. These observations also suggest a key role of MC OXTR signaling in maintaining adult hippocampal NSPC homeostasis. Notably, recent studies showed that Shh signaling originating from MCs is vital for maintaining NSPC pools and preventing their exhaustion due to excessive consumption in aging and after seizures [56, 57]. Future studies are required to determine whether Shh signaling contributes to the maintenance of NSPC pools mediated by MC OXTR signaling.

Despite its significant contribution to understanding the role of MC OXTR signaling in regulating AHN and cognitive functions, this study has several limitations. First, this study used only *Crlr*-Cre-mediated *Oxtr* cKO mice as test subjects. Future studies using other MC-specific transgenic mouse lines (e.g., *Drd2*-Cre) or direct hilar injections of Cre-dependent viral vectors may help address this issue. Second, this study used only male mice as test subjects. Further studies are needed to determine if our findings can be applied to female mice. Third, no pharmacological strategies were used to activate OXTR signaling, leaving it unclear whether exogenous OXT administration could enhance AHN and improve contextual discrimination. Fourth, it still leaves open the question of whether MC OXTR signaling regulates the AHN process through direct glutamatergic or indirect GABAergic pathways. Finally, while it is conceivable that AHN contributes to the DG's pattern separation function in contextual discrimination, future research should investigate whether MC OXTR signaling-mediated regulation of AHN is causally related to contextual discrimination. Restoring impaired AHN caused by MC *Oxtr* deletion during the contextual discrimination test could provide deeper insight into the causal relationship between them. These questions warrant further investigation.

## Conclusions

We have demonstrated that MC *Oxtr* deletion impairs AHN and the ability to discriminate between contexts. Our study highlights the regulation of AHN by local neural circuits [19] and supports the idea that endogenous OXTR signaling indirectly regulates AHN in a non-cell-autonomous manner [22, 58]. Given the prominent role of OXTR signaling in enhancing AHN and preserving NSPC pools, the translational relevance of our work lies in the potential for OXT to serve as a new therapeutic strategy for treating neurodegenerative diseases.

## Data Availability

The authors confirm that all data generated and analyzed during this study are either included in this published article or available from the corresponding authors upon reasonable request.

## Abbreviations

AHN: Adult hippocampal neurogenesis
ANOVA: Analysis of variance
BrdU: 5-Bromo-2'-deoxyuridine
Calb: Calbindin
Crlr: Calcitonin receptor-like receptor
DAPI: 4',6-Diamidino-2-phenylindole
DCX: Doublecortin
DG: Dentate gyrus
DGC: Dentate granule cell
DPI: Days post-infection
FISH: Fluorescent in situ hybridization
GFAP: Glial fibrillary acidic protein
GFP: Green fluorescent protein
MC: Mossy cell
NeuN: Neuronal nuclear protein
NIH: National Institutes of Health
NSPC: Neural stem/progenitor cell
OXT: Oxytocin
OXTR: Oxytocin receptor
PBS: Phosphate-buffered saline
PCR: Polymerase chain reaction
PFA: Paraformaldehyde
SEM: Standard error mean
SGZ: Subgranular zone
SMP: Significant motion pixel
SOX2: SRY-box transcription factor 2
